# Supplementary material for: Photografted Zwitterionic Hydrogel Coating Durability for Reduced Foreign Body Response to Cochlear Implants
Source: ACS Appl Bio Mater. 2024 Apr 8;7(5):3124–35. doi: 10.1021/acsabm.4c00156 (PMC11110053; doi:10.1021/acsabm.4c00156)
Supplement: Supplementary file 1 — mt4c00156_si_001.pdf [file mt4c00156_si_001.pdf]

Supplementary Information for "Photografted zwitterionic hydrogel coating durability for reduced foreign body response to cochlear implants"

Adreann Peel[1], Douglas Bennion[2], Ryan Horne[1], Marlan R. Hansen[2], C. Allan Guymon[1*]

1. Department of Chemical and Biochemical Engineering, University of Iowa, Iowa City, Iowa 52242 USA

2. Department of Otolaryngology-Head and Neck Surgery, University of Iowa, Iowa City, Iowa 52242 USA

*Corresponding author, allan-guymon@uiowa.edu

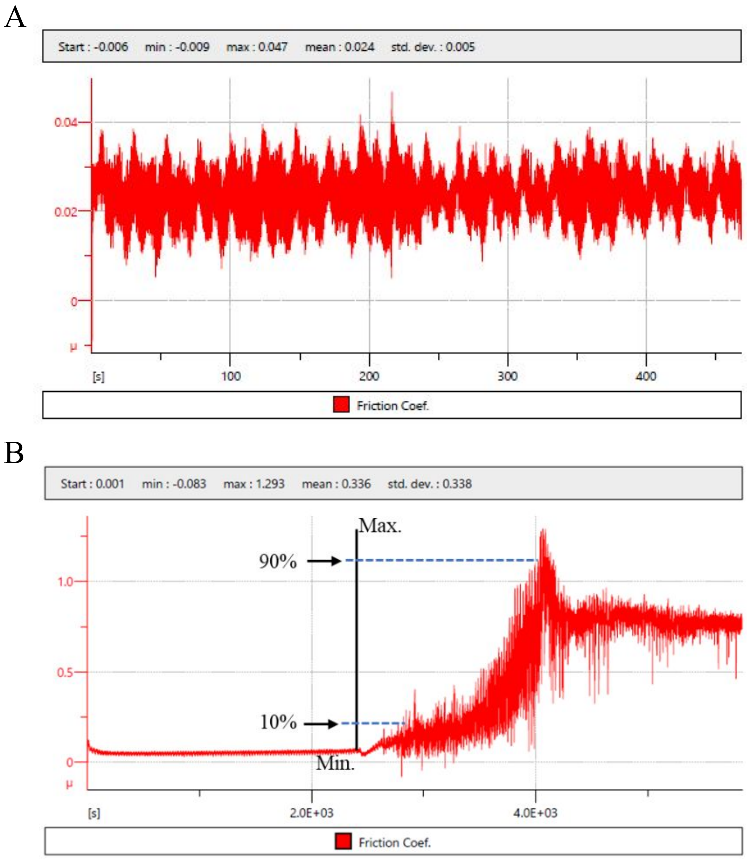


**Figure S1.** Representative tribometer curves for SBMA hydrogels which are A) fully hydrated for the duration of measurement and B) exposed to ambient conditions and allowed to desiccate from the initial hydrated state. B) indicates the general trend during desiccation and how T10 and T90 values were calculated.

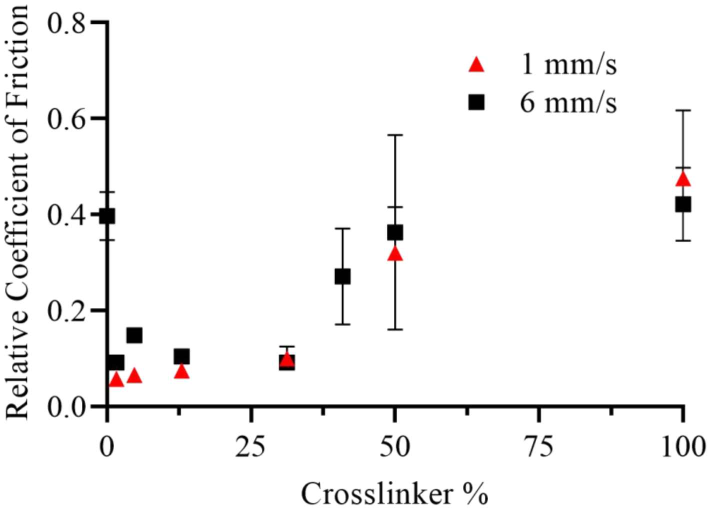


**Figure S2.** Relative coefficient of friction for SBMA hydrogels as a function of crosslink density for two probe speeds.
